# Immunological Aspects of Gastrointestinal Diseases

**DOI:** 10.1155/2017/2891574

**Published:** 2017-05-17

**Authors:** Shahram Golbabapour, Luísa M. da Silva, Antonios Athanasiou

**Affiliations:** ^1^Shiraz Transplant Research Centre, Shiraz University of Medical Sciences, Shiraz, Iran; ^2^Centre of Health Sciences, Faculty of Pharmacy, University of Itajai, Itajaí, Brazil; ^3^Department of Surgery, Mercy University Hospital, Cork, Ireland

The gastrointestinal tract is an important organ that directly and indirectly interacts with various organs in the body to produce energy and basic nutrients from food intake. Diseases of gastrointestinal tract not only have various effects on its several functions but endanger patients through its effects on the other organs. Therefore, the maintenance of homeostatic state of this organ is critically important for remaining healthy. In addition to the main physical and chemical functions of the gastrointestinal tract, this organ is a habitat for various microorganisms named microflora which has beneficial functions through a mutually beneficial association with the body. Autoimmune diseases of gastrointestinal system appear with high incidence among adult and children. Primary sclerosing cholangitis is a cholestatic liver disease that causes chronic inflammation of biliary duct. The pathogenesis of this disease is previously well-explained by Pollheimer et al. [1] and Eaton et al. [2]. According to recent research about the role of genetic and immune factors involved in the pathogenesis, primary sclerosing cholangitis appeared to be a common disease among children. Similarly, autoimmune pancreatitis which is a chronic pancreatitis with a high prevalence is one of the important gastrointestinal diseases whose diagnosis is mainly based on immunological parameters [3]. Genetic factors are the main causes of this disease as previously described by Whitcomb [4]. Another chronic inflammation disease in gastrointestinal tract is bowel disease in which the level of interleukin 17 significantly increases [5]. Moreover, host-microbe interaction is crucially important in the incidence of the disease [6] and may attribute in HIV replication as comprehensively reviewed by Brenchley and Douek [7]. The main focus of this special issue is on recent improvements in the immunological aspects of gastrointestinal diseases. Furthermore, gastrointestinal functions and pathophysiology features of gastrointestinal diseases are also reviewed in this special issue.

In the paper entitled “The Role of Genetic and Immune Factors for the Pathogenesis of Primary Sclerosing Cholangitis in Childhood,” P. M. Ferri et al. reviewed the effects of immune and genetic factors in the pathogenesis of primary sclerosing cholangitis (PSC) in paediatric patients. The authors discussed that human leukocyte antigen (HLA) class I and class II are the main risk factors for PSC in major histocompatibility complex. Moreover, changes in immune response to pathogens, activation of T lymphocytes, and the release of inflammatory mediators and adhesion molecules contribute to the pathogenesis of PSC as well.

In a review paper entitled “From Pathogenesis, Clinical Manifestation, and Diagnosis to Treatment: An Overview on Autoimmune Pancreatitis,” O. Cai and S. Tan discussed the types of autoimmune pancreatitis (AIP) and its classification. Pathogenesis, clinical manifestation, and differential diagnosis of the disease are also discussed in this review paper. Besides, the treatment of AIP disease is elaborated in brief.

In this special issue, another review article entitled “The Immunological Basis of Inflammatory Bowel Disease” is published. In this work, F. Silva et al. discussed microbiota and genetic factors involved in bowel disease. Moreover, the roles of immune system including innate and adaptive immunities are summarized in brief. The main cytokines of the adaptive immune response, the effector cells, and their principle actions are explained separately. The importance of T regulatory cells and helper 17 cells in bowel disease is highlighted.

In the paper entitled “The Value of Caspase-3 after the Application of *Annona muricata* Leaves Extract in COLO-205 Colorectal Cancer Cell Line,” M. Abdullah et al. evaluated the apoptosis-inducing effect of soursop leaf extract (*Annona muricata*) in COLO-205, a colorectal cancer cell line, through the activities of caspase-3, which is a marker of cell apoptosis. In this study, *Annona muricata* leaf extract showed a better level of caspase-3 as compared with leucovorin (folinic acid) and placebo. The authors concluded that this result may suggest that leaf extract of *Annona muricata* has anticancer properties through enhancing the activity of caspase-3.

In the paper entitled “Investigation of Small Bowel Abnormalities in HIV-Infected Patients Using Capsule Endoscopy,” E. Sakai et al. assessed the amendments in intestinal mucosal among HIV patients using capsule endoscopy. The authors concluded that HIV infection itself is able to induce injuries in small bowel mucosal. However, such injuries seem unlikely to cause major clinical symptoms. In addition, HIV infection is closely associated with villous atrophy, which is not completely restored even after the measure of highly active antiretroviral therapy.
